# Entomological Assessment of the Status and Risk of Mosquito-borne Arboviral Transmission in Ghana

**DOI:** 10.3390/v12020147

**Published:** 2020-01-27

**Authors:** Michael Amoa-Bosompem, Daisuke Kobayashi, Katsunori Murota, Astri Nur Faizah, Kentaro Itokawa, Ryosuke Fujita, Joseph Harold Nyarko Osei, Esinam Agbosu, Deborah Pratt, Shohei Kimura, Kofi Dadzie Kwofie, Mitsuko Ohashi, Joseph H. Kofi Bonney, Samuel Dadzie, Toshinori Sasaki, Nobuo Ohta, Haruhiko Isawa, Kyoko Sawabe, Shiroh Iwanaga

**Affiliations:** 1 Department of Environmental Parasitology, Tokyo Medical and Dental University, 1-5-45 Yushima, Bunkyo-ku, Tokyo 113-8510, Japan; mamoabosompem@gmail.com (M.A.-B.); Sho.12.kim@gmail.com (S.K.); kwofiek@gmail.com (K.D.K.); mitsukoohashi0605@gmail.com (M.O.); 2 Department of Medical Entomology, National Institute of Infectious Diseases, 1-23-1 Toyama, Shinjuku-ku, Tokyo 162-8640, Japan; dkoba@nih.go.jp (D.K.); astrinf@nih.go.jp (A.N.F.); tsasaki@nih.go.jp (T.S.); sawabe@nih.go.jp (K.S.); 3 Department of Parasitology, Noguchi Memorial Institute for Medical Research, University of Ghana, College of Health Sciences, P.O. box LG 581, Legon, Accra, Ghana; JOSEI@noguchi.ug.edu.gh (J.H.N.O.); SDadzie@noguchi.ug.edu.gh (S.D.); 4 Kyushu Research Station, National Institute of Animal Health, NARO, 2702 Chuzan, Kagoshima 891-0105, Japan; k.murota@affrc.go.jp; 5 Graduate School of Agricultural and Life Science, The University of Tokyo, 1-1-1 Yayoi, Bunkyo-ku, Tokyo 113-8657, Japan; 6 Pathogen Genomics Center, National Institute of Infectious Diseases, 1-23-1 Toyama, Shinjuku-ku, Tokyo 162-8640, Japan; itokawa@nih.go.jp; 7 Laboratory of Sanitary Entomology, Kyushu University Graduate School of Bioresource and Bioenvironmental Sciences, 744 Motooka, Nishi-ku, Fukuoka 819-0395, Japan; r-fujita@agr.kyushu-u.ac.jp; 8 Department of Virology, Noguchi Memorial Institute for Medical Research, University of Ghana, College of Health Sciences, P.O. box LG 581, Legon, Accra, Ghana; eagbosu@noguchi.ug.edu.gh (E.A.); dpratt@noguchi.ug.edu.gh (D.P.); kbonney@noguchi.ug.edu.gh (J.H.K.B.); 9 Faculty of Health Science, Suzuka University of Medical Science, 1001-1 Kishioka-cyo, Suzuka-shi, Mie 510-0293, Japan; nohta@suzuka-u.ac.jp

**Keywords:** *Aedes aegypti*, *Culex spp*., cryptic species, dengue virus, mosquito virome, insect-specific virus, totivirus, Ghana, virus-virus interaction

## Abstract

Entomological surveillance is one of the tools used in monitoring and controlling vector-borne diseases. However, the use of entomological surveillance for arboviral infection vector control is often dependent on finding infected individuals. Although this method may suffice in highly endemic areas, it is not as effective in controlling the spread of diseases in low endemic and non-endemic areas. In this study, we examined the efficiency of using entomological markers to assess the status and risk of arbovirus infection in Ghana, which is considered a non-endemic country, by combining mosquito surveillance with virus isolation and detection. This study reports the presence of cryptic species of mosquitoes in Ghana, demonstrating the need to combine morphological identification and molecular techniques in mosquito surveillance. Furthermore, although no medically important viruses were detected, the importance of insect-specific viruses in understanding virus evolution and arbovirus transmission is discussed. This study reports the first mutualistic relationship between dengue virus and the double-stranded RNA Aedes aegypti totivirus. Finally, this study discusses the complexity of the virome of *Aedes* and *Culex* mosquitoes and its implication for arbovirus transmission.

## 1. Introduction

Arboviruses are generally regarded as agents of emerging or re-emerging diseases [1]. Arboviral infections can be asymptomatic and self-limiting but can also cause flu-like symptoms, neurological defects, and in some cases, death [2]. As the classification suggests, the distribution and intensity of arboviral infections differ around the world. The mosquito-borne arboviruses, Yellow fever virus (YFV), dengue virus (DENV), Zika virus (ZIKV), West Nile virus (WNV), and chikungunya virus (CHIKV), are globally distributed, whereas the Japanese encephalitis virus, Murray Valley encephalitis virus, and Rift Valley fever virus have a more regional distribution [3,4]. Arboviruses are transmitted by arthropods, and there are about 300 types of mosquitoes capable of transmission. However, the *Aedes* and *Culex* mosquitoes are considered the most medically important mosquito vectors [5].

Climate change and globalization directly impact the spread of mosquitoes and are generally considered major factors influencing the transmission of arboviruses [3]. *Aedes aegypti*, for example, is one of the primary mosquito vectors of arboviruses that are globally distributed and is known to have originated in Africa, whereas *Aedes albopictus*, which has been implicated as a mosquito vector in parts of the world, was imported into Africa about 30 years ago [6].

The sylvatic cycle describes the maintenance of the arboviral life cycle in wild animals that include, but are not limited to, non-human primates and ensures the continuous survival of the virus between epidemics [5,7]. Arboviruses colonize and remain dormant in mosquito eggs, which hatch into adult female mosquitoes that are already infected when conditions are favorable, aiding the spread of arbovirus endemicity [5]

Due to the close interaction between mosquito vectors and arboviruses, the spatial distribution of a vector has been used to estimate the risk of arboviral infection or outbreak, as well as the population at risk (PAR) of infection, particularly in Africa, where there are gaps in the information pool [6]. In 2015, 831 million people in Africa were estimated to be at risk of infection with at least one of the four major *Aedes* transmitted arboviruses: YFV, DENV, ZIKV, and CHIKV [6]. Although these estimates may affect policy and help prevent outbreaks, they also draw attention to the absence of information on the true disease burden. Nonetheless, the collection of information on the species and sub-species of mosquitoes located in each country/region remains crucial in vector control.

In an attempt to fully understand and appreciate the role of the vector in disease transmission, recent studies have focused on how the virome of the mosquito, especially insect-specific viruses (ISVs), affects arbovirus-mosquito interactions and arboviral transmission [8,9,10,11,12,13]. Though recent studies have enhanced our understanding of the evolutionary history of medically important arboviruses, data on the virome of mosquitoes in general, particularly in Africa, remains limited [6]. Further exploration of the mosquito-virome is warranted because of the significant differences in the relative abundance and intensity of outbreaks in different geographical areas, with Africa recording the lowest numbers.

For example, with the exception of YFV outbreaks in the past, which are currently under control because of active vaccination [6], there have been no arboviral outbreaks in Ghana, despite a PAR estimate that identified Ghana as one of the high-risk zones in West Africa [6]. That being said, a large number of patients with undiagnosed febrile conditions are reported in Ghana each year [6,14], raising concerns that in some cases, these illnesses may be due to arboviral infections.

This study used entomological tools to assess the status and risk of arboviral infections in Ghana. This study also investigated the diversity and complexity of the virome of *Aedes* and *Culex* mosquitoes, including their evolutionary histories.

This study was conducted in two phases; the first was a pilot study in the capital city of Ghana, the Greater Accra Region, between September 2015 and October 2015 and focused primarily on adult *Aedes* and *Culex* mosquitoes. The second phase was a larger cross-sectional study conducted in six regions, across five different vegetations, in Ghana between July 2016 and August 2016. This element of the study included all phases of the mosquito life cycle and all species of mosquitoes including those found in Ghana’s largest wildlife refuge, Mole National Park.

Finally, since the sample collection period overlapped with the DENV outbreaks in Cote d’Ivoire in 2015 and in Burkina Faso in 2016, this study gives an idea of the status of DENV in Ghana during the outbreak and reports on the first mutual interaction between DENV and a double-stranded RNA (dsRNA) virus.

## 2. Materials and Methods

### 2.1. Collection Site

**Figure 1:**
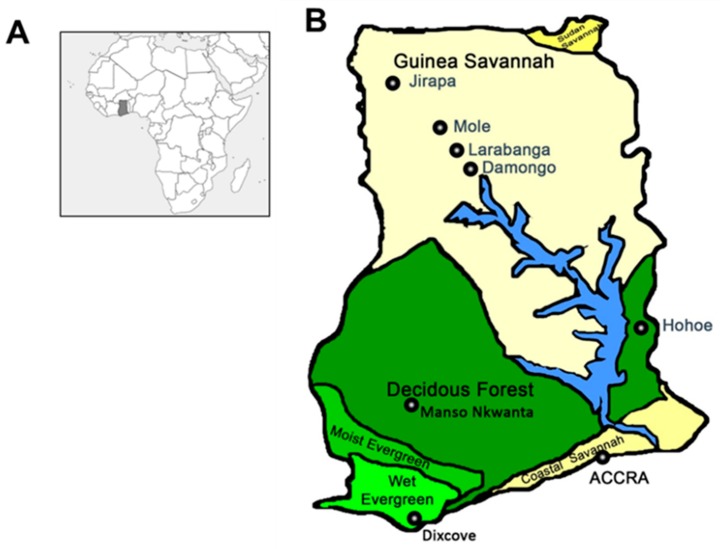
Map of mosquito collection sites. (**A**) Map of Africa showing the location of Ghana (shaded region). (**B**) Map of Ghana showing the collection sites. The collection sites can be divided into 6 Regions (and 5 vegetations); Greater Accra Region (Coastal Savannah); Accra, Volta Region (Forest); Hohoe, Ashanti Region (Forest); Manso Nkwanta, Western Region (Wet Evergreen); Dixcove, Savannah Region (Guinea Savannah); Damongo, Larabanga and Mole National Park and Upper West Region (Guinea Savannah); Jirapa.

Mosquito surveillance was conducted in six regions in Ghana. Ghana is north of the equator on the Gulf of Guinea and has a warm tropical climate with two distinct seasons (dry and wet). The South of Ghana is warm and humid, whereas the North of Ghana is hot and dry. The rainy season runs from March to November and from July to September in the south and north of Ghana, respectively. Mosquito surveillance was conducted in four regions in the south of Ghana (Greater Accra, Volta, Western, and Ashanti regions) and two regions in the north of Ghana (Savannah and Upper West regions). The mosquito surveillance was conducted from September 2015 to October 2015 and July 2016 to August 2016 (Figure 1; Table S1).

### 2.2. Larval Collection, DNA Barcoding, and the Establishment of Laboratory Colonies

Mosquito larvae were collected from water puddles, artificial containers, tree holes, manholes, gutters/waste channels, and used tires. Larval mosquitoes were transferred to the Noguchi Memorial Institute for Medical Research and reared to adults. Once emerged, adult mosquitoes were morphologically identified using taxonomic keys [15,16] and were pinned as voucher specimens and stored at the Department of Medical Entomology, National Institute of Infectious Diseases (NIID), Japan. A leg was removed from the dry-pinned specimens, and DNA was extracted using the alkaline method [17]. The DNA barcoding region of cytochrome c oxidase subunit I was amplified using the universal primers LCO1490 and HCO2198 [18]. The PCR reaction was performed with PrimeSTAR Max DNA polymerase (Takara Bio, Shiga, Japan) under the following conditions: 35 cycles of 98 °C for 10 s, 55 °C for 5 s, and 72 °C for 5 s. PCR products were purified from agarose gels after size verification and sequenced using the 3130 Genetic Analyzer (Applied Biosystems, Waltham, MA). All sequences were submitted to the NCBI database (Table S2).

Prior to morphological identification and DNA barcoding, *Aedes* mosquito larvae collected from four different regions (Greater Accra, Volta, Savannah, and Upper West) were reared to adults, blood fed and allowed to lay eggs. Their progeny (F_1_) was used to establish four different laboratory colonies: GH 23 (Hohoe, Volta Region), GH 98 (Larabanga, Savannah Region), GH 106 (Jirapa, Upper West Region), and GH 115 (Tesano, Greater Accra Region). The second-generation (F_2_) post larval collection was subjected to virome analysis.

### 2.3. Adult Mosquito Collection

Adult mosquito collection was mostly carried out from tire storage areas in car repair shops and a wildlife refuge in Northern Ghana. The collection was performed using sweep nets between 3 pm and 6 pm and CO_2_-baited mosquito traps overnight [19]. The collections were carried out from September 2015 to October 2015 and July 2016 to August 2016. The collected mosquitoes were classified into species, genus, and sex [16,17]. The identified mosquitoes were stored at −80 °C prior to being used for virus detection and isolation.

### 2.4. Virus Isolation from Wild Mosquitoes

Virus isolation was performed using a previously described method with modifications [20]. A maximum of 36 mosquitoes per pool were homogenized in Eagle’s minimum essential medium (Sigma-Aldrich, St. Louis, MO); supplemented with 2% heat-inactivated fetal bovine serum (Sigma-Aldrich) and non-essential amino acids (NEAA, Sigma-Aldrich), 200 U/mL penicillin (Life Technologies, Carlsbad, CA), 200 μg/mL streptomycin (Life Technologies), 5 μg/mL fungizone (Life Technologies); and filter sterilized. The filtrate was used as the inoculum for virus isolation, and the residue was screened for medically important mosquito-transmitted arboviruses.

Virus isolation was performed by inoculating the filtrate onto a monolayer of C6/36 mosquito cell lines (European Collection of Authenticated Cell Cultures) or Vero cells (African green monkey kidney, American Type Culture Collection) and by incubating it at 28 °C/5% CO_2_ and 37 °C/5% CO_2_, respectively, for 7 days. Each isolate was sub-cultured twice in blind passages and supernatants stored at −80 °C prior to being used [21].

### 2.5. Detection of Medically Important Mosquito-transmitted Arboviruses 

To determine the transmission status of medically important mosquito-transmitted arboviruses in Ghana, *Aedes* and *Culex* mosquito pools were screened for DENV, CHIKV, or WNV by RT-PCR. Screening was carried out using the PrimeScript One Step RT-PCR Kit ver. 2 (Takara Bio) and the species-specific primers DENV (D1: 5’-TCAATATGCTGAAACGCGCGAGAAACCG-3’ and D2: 5’-TTGCACCAACAGTCAATGTCTTCAGGTTC-3’); CHIKV (Chik10294s, 5’-ACG CAA TTG AGC GAA GCA CAT-3’ and Chik10573c, 5’-AAA TTG TCC TGG TCT TCC TG-3’ for CHIKV); and WNV (WNNY514, 5’-CGG CGC CTT CAT ACA CW-3’ and WNNY904, 5’-GCC TTT GAA CAG ACG CCA TA-3’ for WNV) [22,23,24]. The cycle conditions used were 50 °C for 30 min, 94 °C for 2 min, and 35 cycles of 94 °C for 30 s, 53 °C for 30 s, and 72 °C for 30 s. The resulting products were analyzed by agarose gel electrophoresis.

### 2.6. Next-Generation Sequencing and Detection of Virus Isolates 

RNA virus detection was performed using the next-generation sequencer (NGS) on cytopathic effect-causing (CPE-causing) supernatants in 2015 and on all supernatants in 2016 [20]. A total of 390 μL of cell supernatant was treated with four units of TURBO DNase (Life Technologies), four units of Baseline zero DNase (Epicentre, Madison, WI), and 0.4 µg of RNase A at 37 °C for 1 h. Nuclease treatment was followed by total RNA extraction using ISOGEN II (Nippon Gene, Tokyo, Japan). The extracted RNA was used to prepare the cDNA library and amplified with the SeqPlex RNA Amplification kit (Sigma-Aldrich) in 2015 and the NEBNext Ultra RNA First Strand Synthesis Module (New England BioLabs, Ipswich, MA) in 2016. The sequence library was prepared from the cDNA library using the ion Plus Fragment library kit (Life Technology) in 2015 and the Truseq Nano DNA LT Library Prep kit (Illumina, San Diego, CA) in 2016. NGS was performed with the Ion PGM in 2015 and Illumina MiniSeq in 2016. Reads were assembled in CLC genomics workbench software (CLC bio, Aarhus, Denmark) and compared with other nucleotide and amino acid sequences by Blastn and Blastx on the NCBI Blast database. Virus positive pools were confirmed by RT-PCR using gene-specific primers designed from each assembled genome.

### 2.7. Genetic Characterization of Detected Viruses 

Gaps found in viral contigs from the NGS data were filled by Sanger sequencing using the ABI 3130 sequencer. Open reading frames (ORFs) in the virus genome were identified using GENETYX ver. 10 software.

### 2.8. Plaque Purification of Isolated Viruses and Complete Genome Determination 

Plaque purification was performed on CPE-causing single-stranded RNA (ssRNA) viruses using previously described protocols, with some modifications [25]. A monolayer of C6/36 cells was inoculated with serially diluted virus stock in a six-well culture plate. The inoculum was removed after virus adsorption, and cells were covered with 1% agarose media. Plaques were harvested after 4 days post inoculation. Plaques were singly suspended in a culture medium to propagate the purified virus. 

NGS analysis was combined with rapid amplification of cDNA end (RACE) to determine the complete genome sequence of plaque-purified viruses. The 5’ and 3’ RACE were performed using the GeneRacer kit (Thermo Fisher Scientific, Waltham, MA) and TaKaRa RNA PCR kit (AMV) ver. 3 (Takara Bio), respectively, following the manufacturers’ instructions.

Double-stranded RNA viruses were purified using cF11 cellulose [21]. The total RNA extracted from virus-infected C6/36 cells was mixed with CF11 cellulose in 16% ethanol in STE buffer (10 mM Tris-HCl, 1 mM EDTA, and 100 mM NaCl) containing 2-mecarptoethanol. After incubation for 1 h, the suspension was washed with 16% ethanol in STE buffer and dsRNA eluted with STE buffer. Subsequently, dsRNA was concentrated using LiCl and ethanol precipitation. NGS analysis was combined with 5’ and 3’ RACE using the DT88 adaptor primer to determine the complete genome sequence of the dsRNA viruses [26].

### 2.9. Purification of the Virus Particle and Structural Protein Analysis 

The plaque-purified ssRNA virus was propagated in C6/36 cells, and the resulting supernatant was concentrated using the YM-50 centrifugal filter unit (Merck Millipore, Darmstadt, Germany). The virus particle was subsequently purified by sucrose gradient centrifugation. The concentrated supernatant was layered on a 20%–70% linear sucrose gradient and centrifuged at 84,000 g overnight. The observation and collection of the viral band were aided by illumination. The harvested layer was dialyzed in TNE buffer (20 mM Tris-HCl, 150 mM NaCl, and 1 mM EDTA, pH 7.4). Analysis of the purified virus particle was done on a 15% SDS-PAGE gel.

### 2.10. Phylogenetic Analysis of RNA Viruses 

Multiple alignments of nucleotide and amino acid sequences were performed using MAFFT 7 online version [27]. The Gblocks program [28] was used for the extraction of conserved domains/sequences of aligned sequences. Finding suitable amino acid substitution models and constructing dendrograms was carried out on MEGA ver. 6 [29]. The maximum likelihood method, with 1000 bootstrap replicates, was used in constructing all the dendrograms. Bootstrap support is indicated by values on branches.

Genbank accession numbers of genome sequences (in parenthesis) used in extracting conserved domains are as follows: CFAV, cell fusing agent virus (LC496857); CAVV, Cavally virus (LC497421), CxFV, Culex flavivirus (LC504568); KoBV, Korle-bu Aedes virus (LC496785); OdV, Odorna virus (LC497422); TeAV, Tesano Aedes virus (LC496784); GoNV, Goutanap virus (LC504569); MoCV, Mole Culex virus (LC505052 and LC505053); WAV, West Accra virus (LC496489), CxPTV, Culex permutotetra-like virus (LC505019); PCLV, Phasi Charoen-like phasivirus (LC498491), APRV, Aedes pseudoscutellaris reovirus (LC496849); AaVV, Aedes aegypti virga-like virus (LC496783); AaTV, Aedes aegypti totivirus (LC496074).

### 2.11. Confirming Vertical Transmission of Viruses in Laboratory Colonies of Aedes Aegypti 

The F3 generation of colony GH 115 was screened for two novel viruses detected in the F2 generation, Aedes aegypti virga-like virus (AaVV) and Aedes aegypti totivirus (AaTV), to confirm persistently infected colonies. Briefly, five pools of 10 female mosquitoes from colony GH 115 were homogenized and tested for AaVV and AaTV by RT-PCR. Furthermore, individual mosquitoes and dissected body parts (the head, thorax, abdomen, legs, wings, and gonads) were screened to determine the virus-infection rate in the colony as well as the dissemination rate.

### 2.12. DENV Superinfection in C6/36 Cells Persistently Infected with AaTV 

A C6/36 cell culture persistently infected with AaTV was established following a previously established protocol, with modifications [30]. Briefly, a monolayer of C6/36 cells was inoculated with AaTV-infected supernatant in a 25 cm^2^ culture flask at 28 °C/5% CO_2_. The inoculum was removed after virus adsorption and cells were covered with fresh culture medium. AaTV-infected cells were sub-cultured weekly. Persistent AaTV infection was confirmed by RT-PCR and immunohistochemistry.

AaTV-infected C6/36 cells were seeded in a six-well plate at 28 °C/5% CO_2_ overnight. The cells were inoculated with the Dengue virus serotype 1 (DENV-1; D1/Hu/Saitama/NIID100/2014) with a multiplicity of infection (MOI) of 0.01 and 1. Cell supernatants were harvested on days 0, 1, 2, 5, 6, and 7 post-inoculation. The detection and quantification of DENV-1 were done by focus-forming assay using mAb4G2-antibody produced from D1-4G2-4-15 mouse hybridoma cells (ATCC HB-112, American Type Culture Collection), Dako EnVision+ System-HRP Labeled Polymer Antimouse, and DAKO Liquid DAB+ Substrate Chromogen System (Agilent Technologies, Santa Clara, CA), following the manufacturer’s instructions. The viral titers were plotted against time. The experiments were run in triplicates, and all experiments were run with controls.

### 2.13. Accession Numbers

The nucleotide sequence accession numbers of viruses and mosquito species detected in this study have been submitted to the International Nucleotide Sequence Database (DDBJ/EMBL/GenBank) as follows: LC496074, LC496489, LC496783-LC496785, LC496848-LC496857, LC497421-LC497422, LC498491-LC498493, LC504568-LC504569, LC505019 and LC505052-LC505055 for viral sequences (Table 1); LC507830-LC507875 for mosquito sequences (Table S2). 

## 3. Results

### 3.1. Larval Collection and DNA Barcoding 

**Figure 2:**
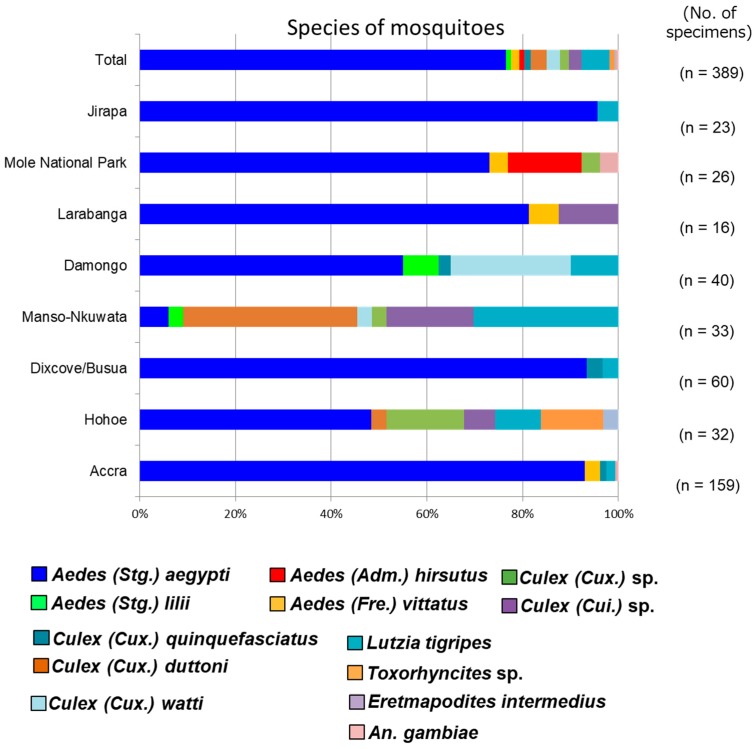
Relative abundance of mosquito species in the collection sites. A total of 13 different species of mosquitoes were detected across 6 regions. Aedes (Stg.) aegypti was the most widely distributed mosquito species across the collection sites. Aedes (Stg.) lilii and Aedes (Adm.) hirsutus were detected for the first time in Ghana.

A total of 401 mosquito larvae were collected in this study across six regions and successfully reared to adults. Out of 401 mosquitoes, 389 were successfully classified into at least 13 different species by combining morphological identification with DNA barcoding (Table S2). Hohoe and Manso-Nkwanta, which are both deciduous forests, had the highest mosquito diversity. Aedes (Stg.) aegypti had the highest distribution across the collection sites, followed by Lutzia tigripes. Aedes (Stg.) lilii, the cryptic species of the yellow fever vector Aedes bromeliae, and Aedes (Adm.) hirsutus, a possible vector of zika virus, were identified for the first time in Ghana (Figure 2).

### 3.2. Adult Mosquito Collection and Detection of Medically Important Arboviruses 

A total of 8680 *Culex* and *Aedes aegypti* mosquitoes were collected over two years. Mosquitoes were divided based on collection sites and pooled based on species and sex. A total of 1138 female mosquitoes divided into fifty pools were screened for medically important arboviruses, while the RNA virome of 188 pools of male and female mosquitoes was analyzed. In this study, we detected and/or isolated 14 RNA viruses belonging to at least eight different families, including the newly proposed taxon Negevirus. No medically important mosquito-transmitted viruses were detected in this study (Table 1). 

### 3.3. Genetic Characterization of RNA Viruses

#### 3.3.1. Negative-Sense ssRNA Virus Phasivirus

**Figure 3:**
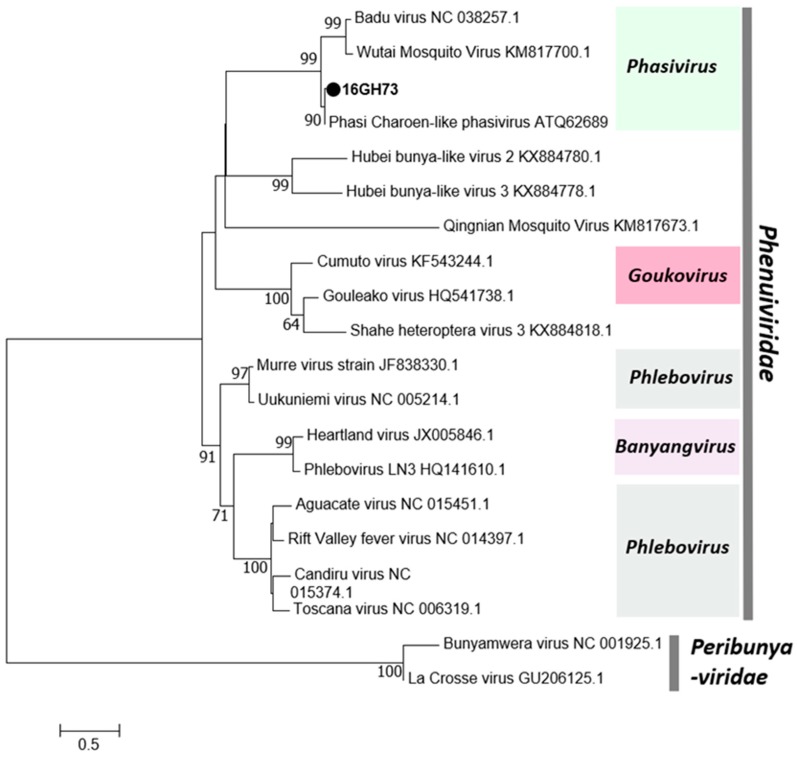
Phylogenetic tree of Phasi Charoen-like Phasivirus (PCLV). Maximum likelihood tree of PCLV constructed with conserved amino acid domains in the RNA-dependent RNA polymerase (RdRp) extracted using MAFFT 7 online version [27] and the Gblocks program [28]. Bootstrap support, from 1000 bootstrap replicates, are indicated by values on the branches. The GenBank accession number of the RdRp, L segment, sequence used is LC498491.

In this study, a single negative-sense RNA virus, Phasi Charoen-like Phasivirus (PCLV), was detected. PCLV was detected in both sexes of *Aedes aegypti* mosquitoes in three regions in 2016. PCLV is one of three reported viruses in the genus *Phasivirus* of the family *Phenuiviridae* and is a tripartite virus with three segments encoding an RdRp (L), a glycoprotein (M), and a nucleocapsid (S). All three segments were detected in this study (Table 1). Consistent with previous reports, the Phasivirus clade was found to be closely related to the unclassified *Phenuiviridae* viruses and the genus Goukovirus (Figure 3). The Gouleako virus, genus Goukovirus, and PCLV have been postulated as basal to all known phleboviruses [31].

#### 3.3.2. Positive-Sense ssRNA Viruses

##### Flaviviruses

**Figure 4:**
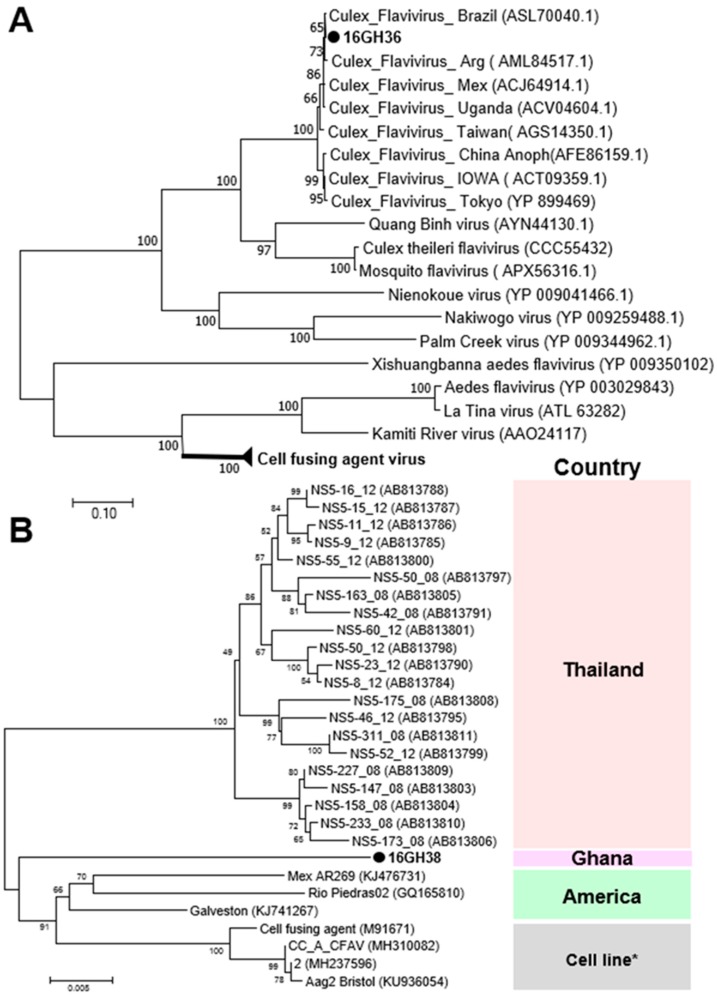
Phylogenetic analysis of Culex flavivirus (CxFV) and cell-fusing agent virus (CFAV). MAFFT 7 online version [27] and the Gblocks program [28] were combined to extract the conserved amino acid domains in CxFV and CFAV genome sequences with Genbank accession numbers LC504568 and LC496857, respectively. The conserved domains were subsequently used in constructing a maximum likelihood phylogenetic tree, with 1000 boostraps, to determine the relationship between CxFV detected in Ghana and other known CxFV and the cell fusing agent virus clade (**A**). The partial nucleotide sequence of NS5 domains of CFAV (Genbank accession number LC496857) was used in determining the phylogenetic position of CFAV from Ghana in relation to previously reported CFAV strains (**B**). Bootstrap support, from 1000 bootstrap replicates, are indicated by values on the branches.

Two ssRNA viruses of the family *Flaviviridae* were detected in the 2016 surveillance. Cell-fusing agent virus (CFAV) was detected in only male and female *Aedes aegypti* mosquitoes collected in the Greater Accra region, whereas Culex flavivirus (CxFV) was found in the Greater Accra and Savannah Regions, but only in female *Culex* mosquitoes (Table 1). Phylogenetic analysis showed both CFAV and CxFV to be more closely related to previously reported strains from the American sub region than those from the Asian region (Figure 4). This may be a direct result of the direction of spread of the vector but may also offer insight into the evolutionary pattern of flaviviruses and other RNA viruses from a single host (ISVs) to a dual host (arboviruses) [32].

##### Alphamesoniviruses

**Figure 5:**
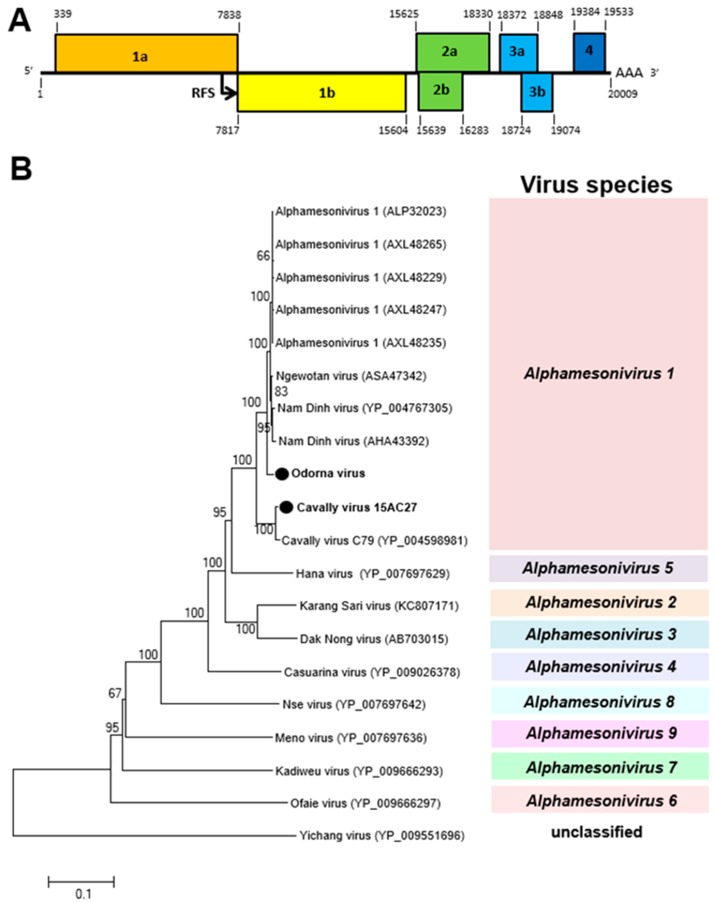
Genetic characterization of Odorna virus (OdV) and Cavally virus (CAVV). (**A**) Genome structure showing ribosomal frameshift (RFS) in the genome. (**B**) The maximum likelihood tree of OdV and CAVV were constructed with conserved amino acid domains in genome sequences, with Genbank accession numbers LC497422 and LC497421, respectively, extracted using MAFFT 7 online version [27] and the Gblocks program [28]. The genetic relationship between OdV, CAVV, and previously reported alphamesoniviruses were shown on the maximum likelihood tree with bootstrap support from 1000 bootstrap replicates indicated by values on branches.

A novel alphamesonivirus, Odorna virus (OdV), and the previously reported Cavally virus (CAVV) were detected in 2016 and 2015, respectively, in the Greater Accra region. OdV was detected in only male *Aedes aegypti* mosquitoes, whereas CAVV was detected in both sexes of *Culex* and *Aedes aegypti* mosquitoes (Table 1). Both viruses belong to the genus *Alphamesonivirus-1* with a ribosomal frameshift (RFS) between ORF 1a and 1b (Figure 5). The conserved slippery sequence GGAUUUU was found in the ORF 1a/1b overlap region [33]. This RFS facilitates the expression of ORF 1b [34].

##### Negeviruses

**Figure 6:**
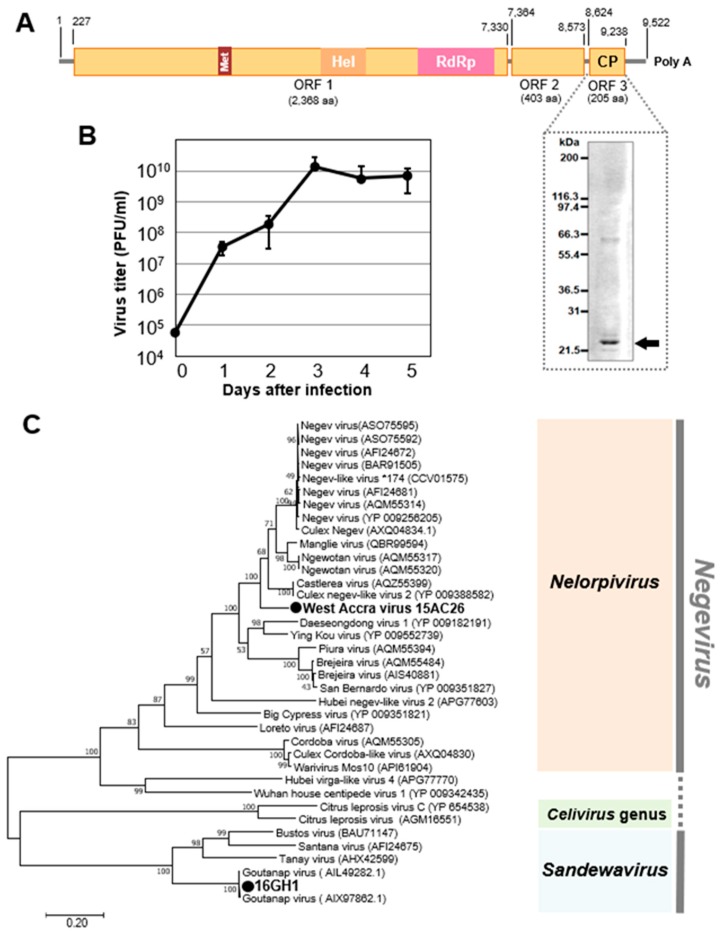
Genetic characterization of West Accra virus (WAV) and Goutanap virus. (**A**) Genome structure of WAV showing the representation of the capsid protein on a PAGE gel. (**B**) Growth rate of WAV in C6/36 cells. (**C**) Phylogenetic relationship between WAV, Nelorpivirus, and Goutanap virus (GoNV), Sandewavirus. The maximum likelihood tree of WAV and GoNV was constructed with conserved amino acid domains in genome sequences with Genbank accession numbers LC496489 and LC504569, respectively. The conserved domains were extracted using MAFFT 7 online version [27] and the Gblocks program [28]. The bootstrap support, from 1000 bootstrap replicates, are indicated by values on the branches.

Two viruses of the Negevirus clade in the genera *Nelorpivirus* and *Sandewavirus* were detected in 2015 and 2016, respectively. West Accra virus (WAV), a novel nelorpivirus, was detected in both sexes of *Culex* and *Aedes aegypti* mosquitoes collected in the Greater Accra region, whereas Goutanap virus, a sandewavirus, was detected in only female *Culex* mosquitoes collected in the Western region (Table 1). Both viruses were found to have three ORFs. ORF 1, the largest ORF, codes for methyl transferase, helicase, and the RdRp. The smallest ORF (ORF 3) in the WAV genome, which is made up of 205 amino acids and codes for the capsid protein, was represented on an SDS-PAGE gel (Figure 6). WAV was found to propagate and cause CPE in C6/36 cells, reaching the log phase of growth within two days of infection (Figure S1).

##### Iflavirus

**Figure 7:**
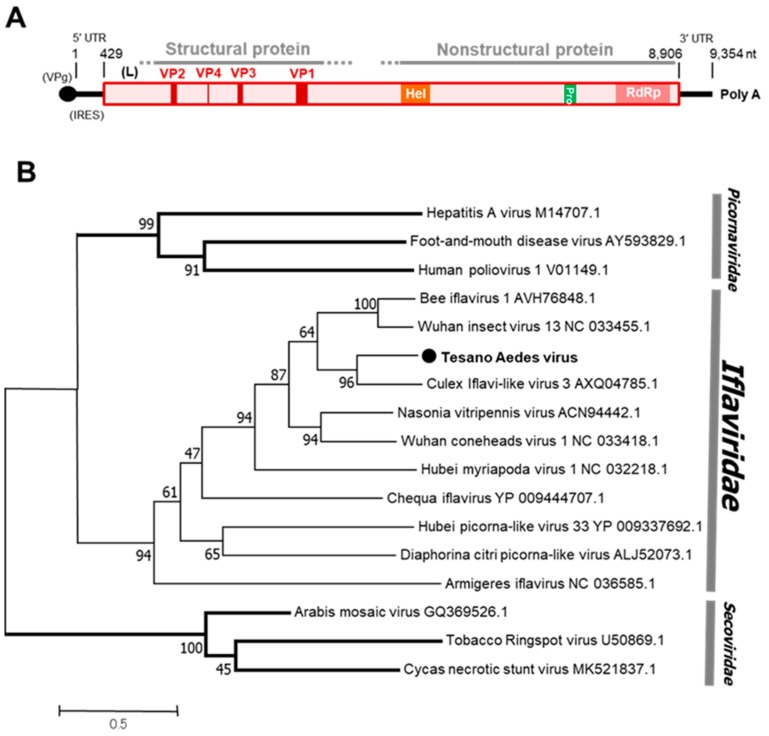
Genetic characterization of Tesano Aedes virus (TeAV). (**A**) Genome structure showing VPg and internal ribosome entry site (IRES) at the 5′ terminal and a poly-A tail at the 3′ terminal. (**B**) Genetic relationship between TeAV and other viruses of the family *Iflaviridae*. The maximum likelihood tree was constructed using the complete amino acid domain of the genome sequence with Genbank accession number LC496784. The amino acid domain was aligned using the MAFFT 7 online version [27]. Bootstrap support values from 1000 bootstrap replicates are indicated on the branches.

Tesano Aedes virus (TeAV), a novel unclassified virus of the family *Iflaviridae*, was detected in both sexes of *Aedes aegypti*, but only in female *Culex* mosquitoes. TeAV was detected in the Greater Accra and Volta regions (Table 1). The genome of TeAV consists of a single ORF with poly-A tract at the 3’ end. The VPg protein and internal ribosomal entry site (IRES), both of which are involved in translation of viral RNA and RNA replication [35], are predicted to be bound to the 5’ end and in the 5’-untranslated region of the viral genome, respectively (Figure 7), which is consistent with related viruses in the family *Iflaviridae*.

##### Tetravirus

**Figure 8:**
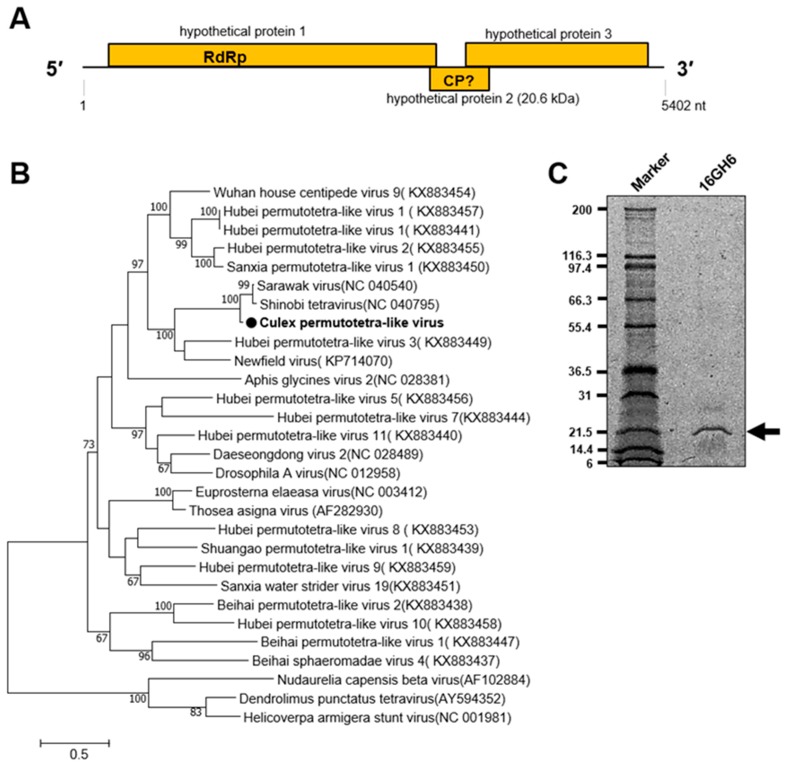
Genetic characterization of Culex permutotetra-like virus (CxPTV). (**A**) Genome structure showing hypothetical protein 2 as possibly coding for the capsid protein. (**B**) Evolutionary history of CxPTV. The maximum likelihood tree was constructed using the complete amino acid domain of the genome sequence with Genbank accession number LC505019. The amino acid domain was aligned using the MAFFT 7 online version [27]. Bootstrap support values from 1000 bootstrap replicates are indicated on the branches. (**C**) Gel representation of CxPTV protein.

A novel tetravirus of the family *Permutotetraviridae* designated Culex permutotetra-like virus (CxPTV) was detected in female *Culex* mosquitoes in the Western and Savannah regions in 2016 (Table 1). The closest related viruses, Sarawak virus and Shinobi tetravirus, were reported to be detected in male *Aedes albopictus* and *Aedes albopictus* cell lines, respectively, whereas the remaining two viruses in the clade were detected in spiders and *Drosophilia melanogaster* [36,37,38,39]. The genome of CxPTV is made up of three ORFs. A 20.6 kDa capsid protein was represented on a PAGE gel and is predicted to be expressed by ORF 2 (Figure 8).

##### Korle-bu Aedes Virus (KoBV)

**Figure 9:**
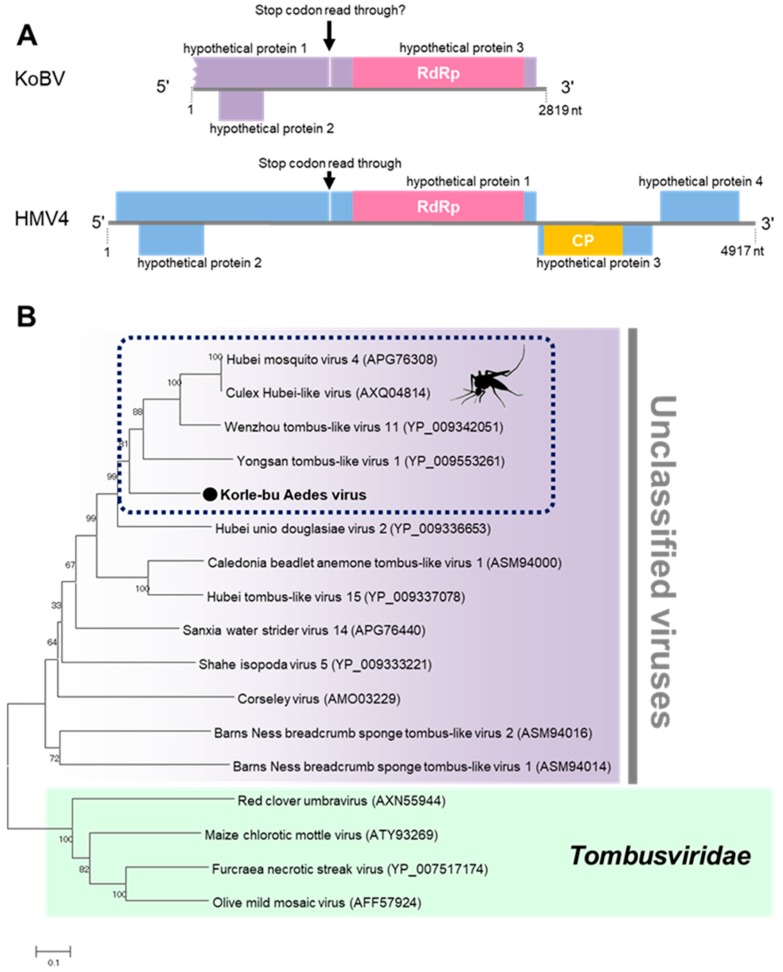
Genetic characterization of Korle-bu Aedes virus (KoBV). (**A**) KoBV genome showing the predicted stop codon read through. (**B**) Evolutionary history of KoBV. Maximum likelihood tree constructed with the conserved amino acid domain of the genome sequence with Genbank accession number LC496785. The conserved domains were extracted using MAFFT 7 online version [27] and the Gblocks program [28]. Bootstrap support values from 1000 bootstrap replicates are indicated on the branches.

Korle-bu Aedes virus (KoBV) is a novel virus that forms a clade with mosquito-associated unclassified viruses closely related to the family *Tombusviridae*. KoBV was detected in female *Aedes aegypti* mosquitoes only in the Greater Accra region in 2016 (Table 1). The genome of KoBV is predicted to have a stop codon read through in ORF 1 because of a premature stop codon (UAG) between nucleotides 1253–1256, similar to reports on Hubei mosquito virus 4 (Figure 9) [37]. The stop codon read through is typically observed when a stop codon is misread or recorded as a sense codon, allowing continuous translation until the next stop codon [40]. The stop codon read through facilitates the expression of several viral and cellular genes, including viral replicases [40,41]. KoBV is therefore predicted to have three ORFs, similar to the unclassified Hubei Mosquito virus 4.

##### Mole Culex Virus (MoCV)

**Figure 10:**
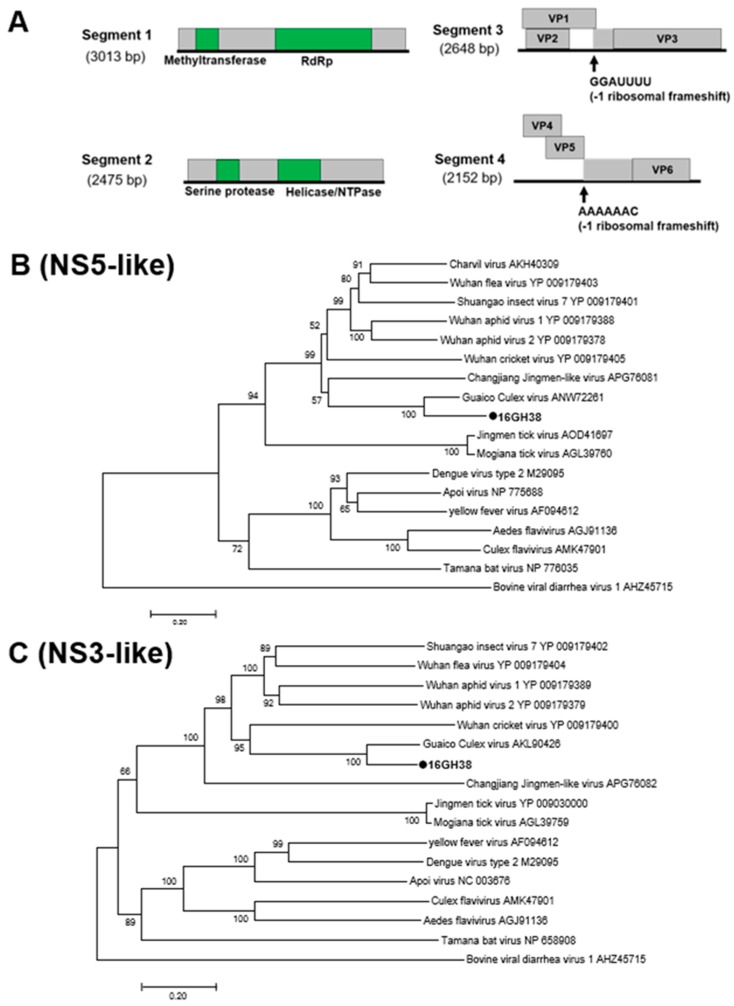
Genetic characterization of Mole Culex virus (MoCV). (**A**) Genome structure of MoCV showing 4 segments and an RFS in segments 3 and 4. (**B**) and (**C**) Phylogenetic analysis using the conserved non-structural (NS) domains. Maximum likelihood trees constructed using the NS3 and NS5 amino acid domains of genome sequences with Genbank accession numbers LC505052 and LC505053, respectively. The amino acid domain was aligned using the MAFFT 7 online version [27]. Bootstrap support values from 1000 bootstrap replicates are indicated on the branches.

Mole Culex virus (MoCV) is a novel multi-component Jingmenvirus detected in female *Culex* mosquitoes collected in the Savannah region in 2016 (Table 1). Jingmenviruses typically have five segments and are closely related to the non-segmented flaviviruses. The fifth and smallest segment is not always present in infections and does not play any role in viral replication [42,43,44]. Consistent with the findings in this study, MoCV has a genome comprised of at least four separately packaged segments (Figure 10) and causes CPE in C6/36 cells (Figure S2). The first two segments were both monocistronic coding for non-structural (NS) domains, similar to the conserved NS5 and NS3 domains in flaviviruses. The remaining segments contained three ORFs each with a predicted RFS. The conserved slippery sequences -GGAUUUU and -AAAAAAC were found in segments three and four, respectively (Figure 10) [43].

##### Aedes Aegypti Virga-like Virus (AaVV)

**Figure 11:**
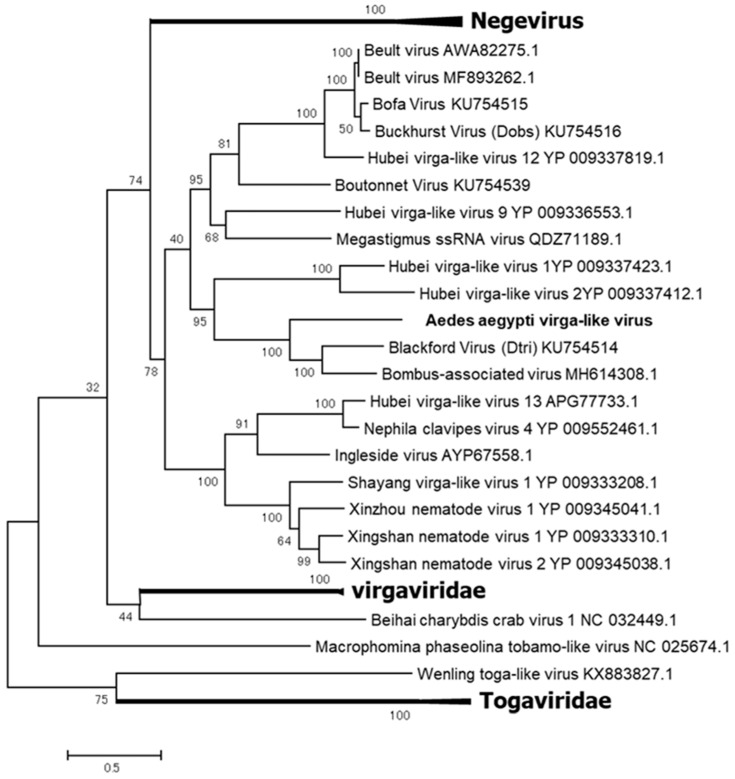
Phylogenetic analysis of Aedes Aegypti virga-like virus (AaVV). Maximum likelihood tree constructed with the complete amino acid domain of the genome sequence with Genbank accession number LC496783. The amino acid domain was aligned using the MAFFT 7 online version [27]. Bootstrap support values from 1000 bootstrap replicates are indicated on the branches.

AaVV is one of two viruses detected in the F_2_ generation of colony GH 115 (Table 1). AaVV has at least three ORFs in its genome and forms a clade with the unclassified viruses closely related to the family *Virgaviridae* and the Negevirus taxon (Figure 11) [45]. AaVV was not successfully transmitted to the F_3_ generation.

#### 3.3.3. Double-stranded RNA Viruses

##### Reovirus

**Figure 12:**
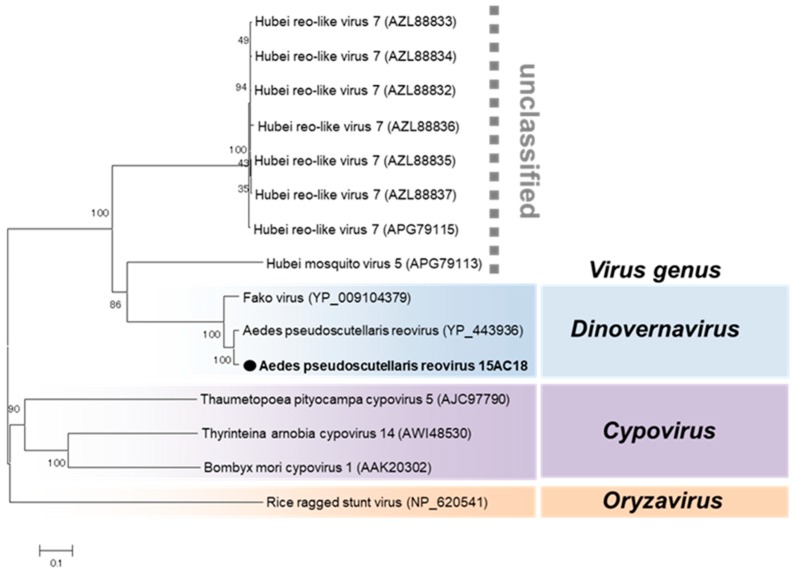
Phylogenetic analysis of Aedes pseudoscutellaris reovirus (APRV). Maximum likelihood tree of APRV constructed with conserved amino acid domains in the RdRp (VP2) extracted using MAFFT 7 online version [27] and the Gblocks program [28]. Bootstrap support values from 1000 bootstrap replicates are indicated on the branches. The GenBank accession number of the RdRp (L segment) sequence used is LC496849.

Aedes pseudoscutellaris reovirus (APRV) was the only virus detected in both 2015 and 2016. APRV was detected in both sexes of *Aedes aegypti* and female *Culex* mosquitoes (Table 1). APRV belongs to the genus *Dinovernavirus* in the family *Reoviridae* (Figure 12). Consistent with the previously reported strain, nine segments of APRV were detected in this study (Table 2). The terminal sequences of all nine segments were determined to be completely identical to the previously reported APRV except segments 6, 8, and 9 which had one nucleotide substitution each (Table 2). As the name suggests, the reported strain of APRV was detected in *Aedes pseudoscutellaris* cell lines, making this the first detection of APRV in wild mosquitoes and *Aedes aegypti* species [46], suggesting a preference for mosquitoes in the genus *Aedes*.

##### Totivirus

**Figure 13:**
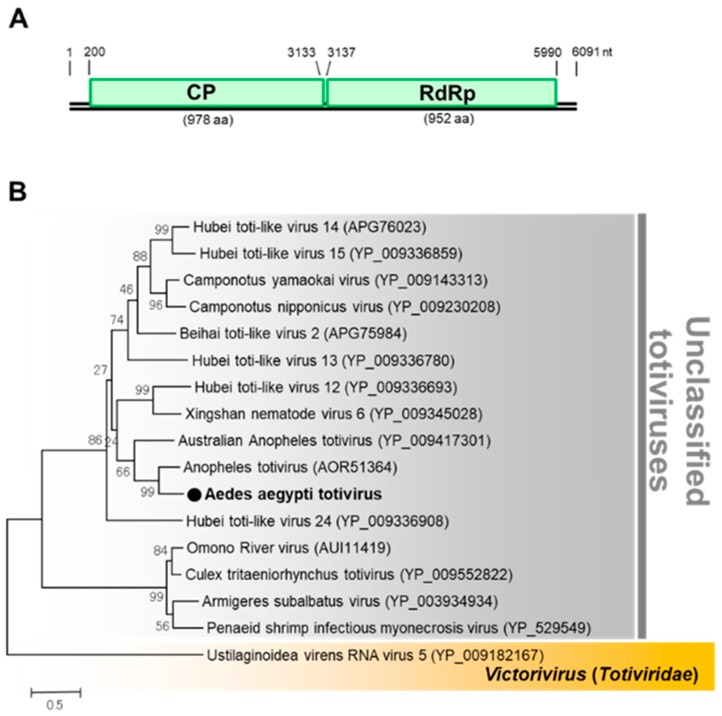
Genomic characterization of Aedes aegypti totivirus (AaTV). (**A**) Genome organization of AaTV. (**B**) Phylogenetic analysis of AaTV. Maximum likelihood tree of AaTV constructed with the conserved amino acid domain of the genome sequence with Genbank accession number LC496074. The conserved domains were extracted using MAFFT 7 online version [27] and the Gblocks program [28]. Bootstrap support values from 1000 bootstrap replicates are indicated on the branches.

Aedes aegypti totivirus (AaTV) was the second virus detected in the F_2_ generation of colony GH115 (Table 1). AaTV is an unclassified virus of the family *Totiviridae*. The genome of AaTV comprises two ORFs encoding a capsid protein and RdRp (Figure 13). AaTV was detected in the F_3_ generation with an infection rate of around 60% confirming successful transmission. AaTV was also detected in all the body parts screened, including the male and female gonads, suggesting trans-ovarian transmission within the colony. A virus-free colony of GH 115 was successfully established. AaTV was also successfully propagated in C6/36, NIID-CTR (*Culex tritaeniorhynchus*) [47], and Ar-13 (*Armigeres subalbatus*) [48] cell lines, but not in MSQ-43 (*Anopheles stephensi*, Malaria Research and Reference Reagent Resource Center) cell line.

### 3.4. DENV Superinfection in C6/36 Cells Persistently Infected with AaTV

**Figure 14:**
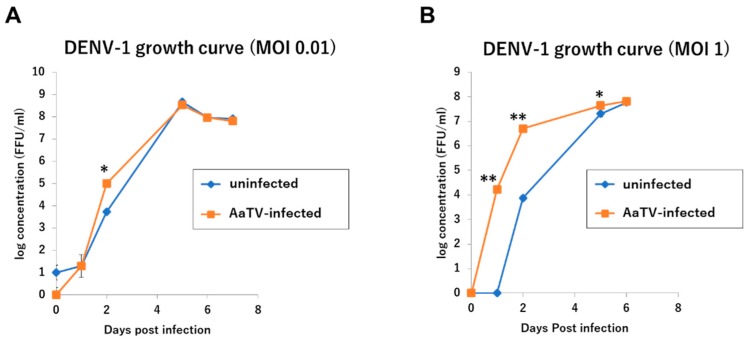
Effect of AaTV infection in C6/36 cells on DENV growth. (**A**) DENV-1 growth curve [multiplicity of infection (MOI) = 0.01] (**B**) DENV-1 growth curve (MOI = 1). Controls were DENV infection on naïve C6/36 cells. Statistical significance was done by the t-test * = *p* ≤ 0.05, ** = *p* ≤ 0.01.

In the determination of the potential effect of AaTV on dengue virus propagation, AaTV-infected C6/36 cells were established and used to propagate DENV-1. AaTV-infected C6/36 cells were observed to boost the initial growth of DENV-1. This effect intensified with increased initial concentrations (MOI) of the DENV-1 virus (Figure 14).

## 4. Discussion

The incidence and distribution of vector-borne arboviruses have increased exponentially over the last 30 years, with migration and globalization regarded as being among the major factors influencing the spread [49]. With a global arboviral PAR of over 40%, active surveillance is an important tool in controlling and preventing the spread of infection. In highly endemic areas, human surveillance serves as a good indicator of the rate of active transmission and as a monitor for the effectiveness of a control measure [49,50]. However, in non-endemic and low endemic areas, human surveillance, which involves taking blood samples, may be considered a non-effective invasive method of disease control. For this reason, vector surveillance, which is monitoring of the presence and density of a disease vector, such as a mosquito vector, in a community is increasingly being explored as a possible tool for assessing the risk of infection [6]. Ghana is regarded as a high-risk country for arboviruses, although it is yet to record any arbovirus outbreak other than yellow fever, which is under control because of active vaccination. Nonetheless, recent reports have noted instances of possible locally acquired infections [51,52,53,54]. Therefore, this study combined entomological surveillance with virus screening and isolation, and RNA virus characterization, to determine the status and risk of arbovirus transmission in Ghana.

Morphological techniques were combined with DNA barcoding to determine the presence and distribution of mosquito species in Ghana. The high density and distribution of *Culex* and *Aedes* mosquitoes in Ghana raise concerns about the possible risk of arboviral outbreaks [55]. The detection of *Aedes hirsutus*, which has been implicated as a vector of ZIKV [56], is an example of the diversity of possible vectors of arboviruses in Ghana. The detection of *Aedes lilii* in Ghana, the non-vector cryptic species of the yellow fever vector *Aedes bromeliae*, affirms the need to combine molecular techniques with morphological identification when using entomological surveillance to estimate the risk of outbreaks [57,58].

Although sample collection for this study overlapped with the DENV outbreaks in Cote d’Ivoire and Burkina Faso in 2015 and 2016, respectively, no medically important arboviruses were detected. This could indicate the absence of active transmission in the sample sites or areas during sample collection [59].

With respect to the virome of *Aedes aegypti* and *Culex* mosquitoes in Ghana, mosquitoes collected in the Greater Accra region had a more diverse virome than mosquitoes from other regions in Ghana. In addition, all four indigenes reported to have possibly acquired infections locally were residents of the Greater Accra region, raising concerns about DENV transmission in the region [53,54]. However, it is necessary to consider the possible role of the detected ISVs in the cycle of events.

PCLV, for example, has been reported to inhibit the propagation of La Crosse virus, an orthobunyavirus, as well as flaviviruses such as ZIKV and DENV in vitro [60]. Furthermore, although CFAV was initially reported to interact mutually with flaviviruses in vitro, this effect is antagonized by PCLV, and recent reports indicate that CFAV interferes with DENV dissemination in vivo [11,60,61]. These reports show the importance of determining and understanding the role of ISVs in arbovirus transmission, in vitro and in vivo, particularly as this study reports the first mutualistic relationship between a medically important flavivirus, DENV, and a dsRNA virus, AaTV. The possible mechanisms behind this observation and potential implications remain to be determined.

The characterization of ISVs helps in understanding the evolutionary history and trends in potentially important and medically important viruses. The discovery of viruses of the genus *Jingmenvirus* (MoCV), for example, in which two segments have a direct evolutionary relationship with non-segmented flaviviruses, suggests that segmented viruses may be the mid-point in the evolution from non-segmented to multipartite viruses, based on the principle of parsimony [62]. Finding and characterizing novel viruses, therefore, is helpful in deepening our understanding of RNA viruses and may influence future interventions that can prevent, or control, arboviral outbreaks.

## Figures and Tables

**Table 1:** Classification and distribution of viruses detected in 2015 and 2016.

Mosquito	Viral Genome	Virus Species	Abbreviation	Virus Family	Virus Genus	Source	Year of Source-Sample Collection	Total No. of Isolates	Region					Accession No.
									Greater Accra	Western	Volta	Savannah	Upper West	
Field-caught mosquitoes	ssRNA (+)	Cell fusing agent virus	CFAV	*Flaviviridae*	*Flavivirus*	*Ae. aegypti* (female and male)	2016	2	2	0	0	0	0	LC496857 (isolate 16GH83)
		Cavally virus	CAVV	*Mesoniviridae*	*Alphamesonivirus-1*	*Cx. quinquefasciatus* (female and male)	2015	44	44	0	0	0	0	LC497421
						*Ae. aegypti* (female and male)								
		Culex flavivirus	CxFV	*Flaviviridae*	*Flavivirus*	*Culex* spp. (female)	2016	3	2	0	0	1	0	LC504568 (isolate 16GH36)
		Korle-bu Aedes virus*	KoBV	unclassified	unclassified	*Ae. aegypti* (female)	2016	8	8	0	0	0	0	LC496785 (isolate 16GH61)
		Odorna virus*	OdV	*Mesoniviridae*	*Alphamesonivirus-1*	*Ae. aegypti* (male)	2016	1	1	0	0	0	0	LC497422 (isolate 16GH83)
		Tesano Aedes virus*	TeAV	*Iflaviridae*	unclassified	*Ae. aegypti* (female and male) *Culex* spp. (female)	2016	21	18	0	3	0	0	LC496784 (isolate 16GH73)
		Goutanap virus	GoNV	unclassified	Negevirus (proposed genus)	*Culex* spp. (female)	2016	1	0	1	0	0	0	LC504569 (isolate 16GH1)
		Mole Culex virus*	MoCV	unclassified	Jingmenvirus (proposed genus)	*Culex* spp. (female)	2016	3	0	0	0	3	0	Segment 1: LC505052 (isolate 16GH38)
														Segment 2: LC505053 (isolate 16GH38)
														Segment 3: LC505054 (isolate 16GH38)
														Segment 4: LC505055 (isolate 16GH38)
		West Accra virus*	WAV	unclassified	Negevirus	*Cx. qunquefasciatus* (female and male)	2015	17	17	0	0	0	0	LC496489
						*Ae. aegypti* (female and male)								
		Culex permutotetra-like virus*	CxPTV	*Permutotetraviridae*	unclassified	*Culex* spp. (female)	2016	5	0	1	0	4	0	LC505019 (isolate 16GH6)
	ssRNA (-)	Phasi Charoen-like phasivirus	PCLV	*Phenuiviridae*	*Phasivirus*	*Ae. aegypti* (female and male)	2016	7	2	0	1	4	0	L segment: LC498491 (isolate 16GH73)
														M segment: LC498492 (isolate 16GH73)
														S segment: LC498493 (isolate 16GH73)
	dsRNA	Aedes pseudoscutellaris reovirus	APRV	*Reoviridae*	*Dinovernavirus*	*Ae. aegypti* (female and male) *Culex* spp. (female)	2015/2016	32	32	0	0	0	0	(Shown in Table 2)
*Ae. aegypti* laboratory colony	ssRNA (+)	Aedes aegypti virga-like virus*	AaVV	unclassified	unclassified	*Ae. aegypti* laboratory colony GH115	2016	NA	NA	NA	NA	NA	NA	LC496783
	dsRNA	Aedes aegypti totivirus*	AaTV	*Totiviridae*	unclassified	*Ae. aegypti* laboratory colony GH115	2016	NA	NA	NA	NA	NA	NA	LC496074

* Novel viruses named in this study. NA: Not applicable.

**Table 2:** Genome characterization of APRV 15AC18 strain and comparison with other dinovernaviruses.

APRV 15AC18							Amino Acid Sequence Identities and Similarity			
					Terminal Sequences (5’-3’)		APRV		FAKV CSW77	
Segment (Accession No.)	Segment Length (bp)	ORF Length (aa)	Protein	Encoded Gene	5’ Terminus	3’ Terminus	Identity (%)	Similarity (%)	Identity (%)	Similarity (%)
1 (LC496848)	3820	1189	VP1	nonstructural protein	**AGUU**U**AA**UUCCC	**UU**GAUCCUA**AGU**	86.8	98.6	81.7	97.6
2 (LC496849)	3752	1233	VP2	RNA-dependent RNA polymerase	**AGUU**A**AA**CCGCC	**UU**GUUUUUA**AGU**	96.4	99.5	89.5	98.5
3 (LC496850)	3732	1202	VP3	major capsid protein	**AGUU**U**AA**AACCC	**UU**UGAUACU**AGU**	95.8	99.4	90.0	99.0
4 (LC496851)	3375	1003	VP4	nonstructural protein	**AGUU**U**AA**AAACC	**UU**AAUCCUA**AGU**	87.4	97.8	72.8	93.3
5 (LC496852)	3227	1056	VP5	turret protein	**AGUU**A**AA**ACCAC	**UU**UAGUAAU**AGU**	95.5	99.7	87.8	98.4
6 (LC496853)	1775	540	VP6	structural protein	**AGUU**U**AA**ACCCA	**UU**UGAUA**A**U**AGU**	94.4	99.6	90.4	98.9
7 (LC496854)	1171	348	VP7	clamp protein	**AGUU**A**AA**AACCA	**UU**UAGUAAU**AGU**	95.7	100.0	87.9	99.1
8 (LC496855)	1151	345	VP8	nonstructural protein	**AGUU**U**AA**AUCC**U**	**UU**UGAUAAU**AGU**	77.7	96.2	64.6	93.3
9 (LC496856)	1151	278	VP9	nonstructural protein	**AGUU**A**AA**ACCCA	**UU**UAGUA**A**U**AGU**	95.7	99.6	80.2	96.8

Underlined sequences are points of nucleotide substitution.

## Supplementary Materials

The following are available online at https://www.mdpi.com/1999-4915/12/2/147/s1, Table S1a: Mosquito pools for virus isolation and virus positive pools in 2015 and 2016. Table S1b: Mosquito collection sites and pools used for virus isolation in 2015. Table S1c: Mosquito collection sites and sampling method used in 2016. Table S2a: Classification of mosquitoes by morphological identification and DNA barcoding. Table S2b: Mosquito species and COI Genbank accession numbers. Figure S1: Phase-contrast micrographs of WAV-infected (A) and mock-infected (B) C6/36 cells at 3 days postinfection. Figure S2: Phase-contrast micrographs of MoCV-infected (A) and mock-infected (B) C6/36 cells at 6 days postinfection.
