# Supplementary material for: Entomological Assessment of the Status and Risk of Mosquito-borne Arboviral Transmission in Ghana
Source: Viruses. 2020 Jan 27;12(2):147. doi: 10.3390/v12020147 (PMC7077231; doi:10.3390/v12020147)
Supplement: Supplementary file 1 [file viruses-12-00147-s001.zip › Supplementary files for Revision/Spplementary Figures.pptx]

## Slide 1
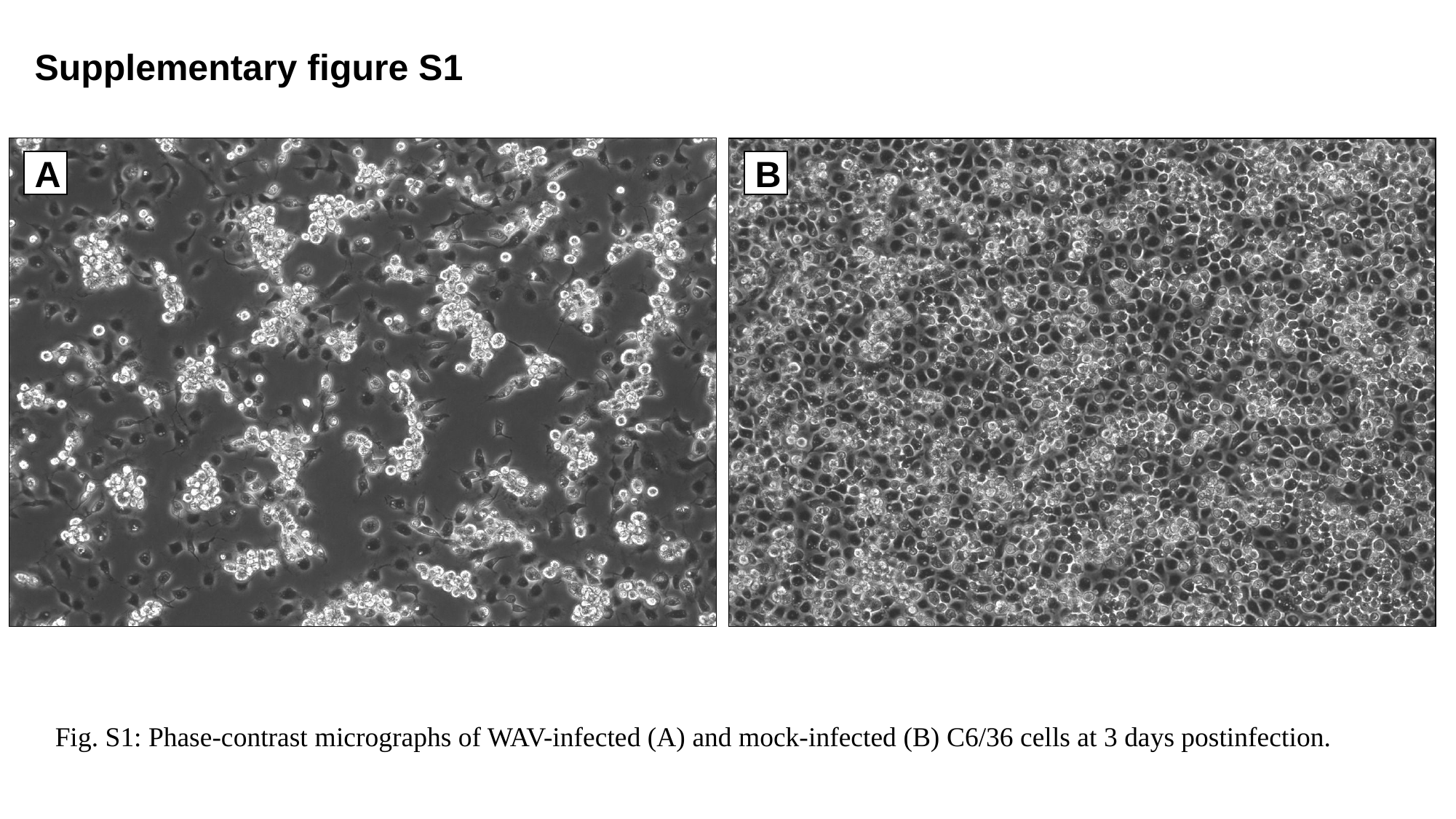

Supplementary figure S1
A
B
Fig. S1: Phase-contrast micrographs of WAV-infected (A) and mock-infected (B) C6/36 cells at 3 days postinfection.

## Slide 2
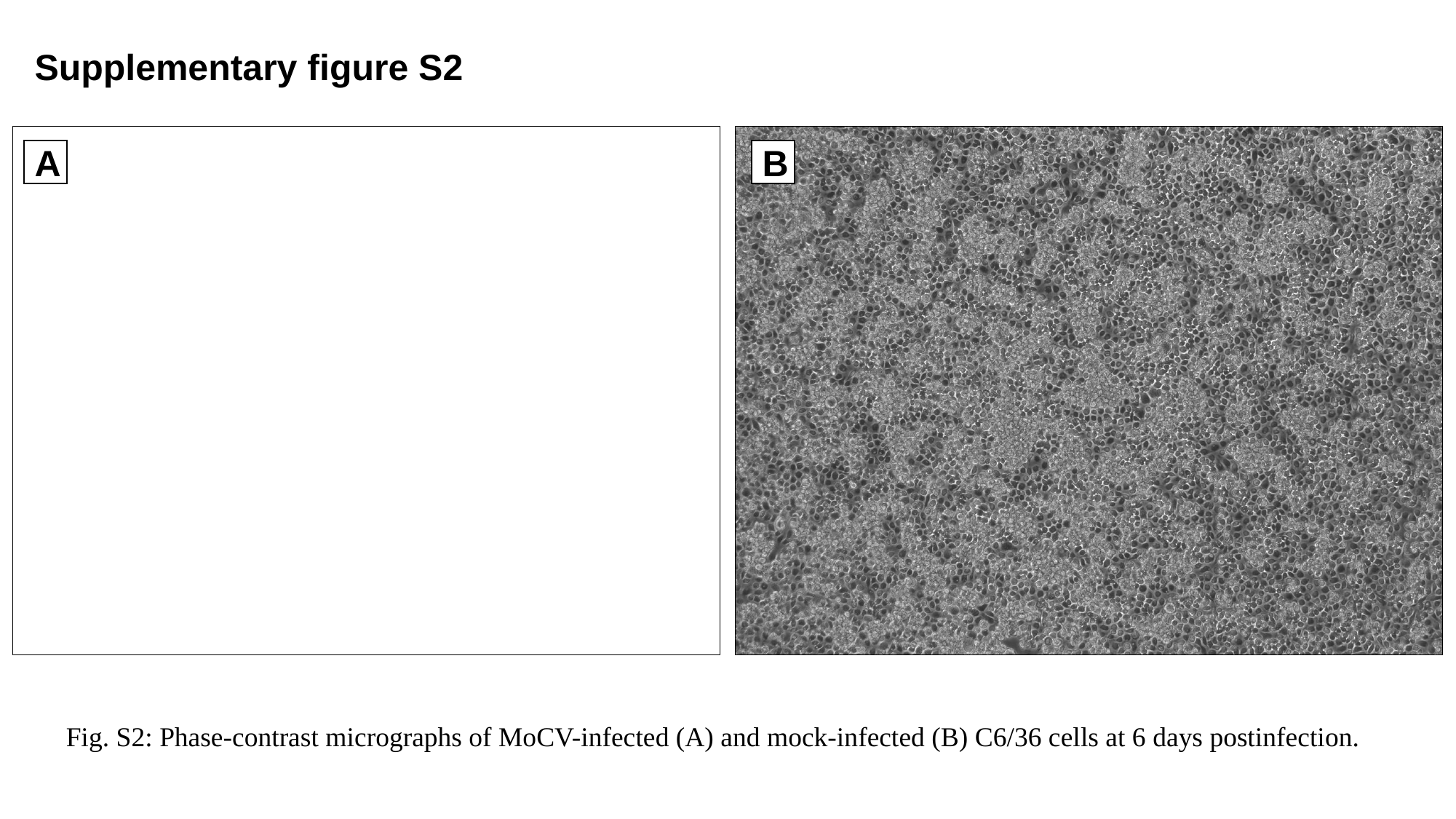

Supplementary figure S2
A
B
Fig. S2: Phase-contrast micrographs of MoCV-infected (A) and mock-infected (B) C6/36 cells at 6 days postinfection.
